# New Onset Diabetes Mellitus Complicated by Hypertriglyceridemia-Induced Pancreatitis

**DOI:** 10.7759/cureus.13569

**Published:** 2021-02-26

**Authors:** Aneeba Farooqi, Yetunde B Omotosho, Farah Zahra

**Affiliations:** 1 Internal Medicine, The Chicago Medical School Internal Medicine Residency Program at Northwestern McHenry Hospital, McHenry, USA

**Keywords:** obesity, obesity and diabetes, hypertriglyceridemic pancreatitis, insulin injection, plasmapheresis, endocrinology and diabetes, lipid disoders

## Abstract

There is an increasing prevalence of type 2 diabetes mellitus (DM) among adolescents due to obesity. Diabetes can cause hypertriglyceridemia, defined as triglyceride (TG) levels above 150 mg/dl, leading to severe complications, including cardiovascular events, fatty liver disease, and acute pancreatitis. We present a case of acute pancreatitis manifested by both hypertriglyceridemia and new-onset DM. The risk of hypertriglyceridemia-induced pancreatitis (HTGP) significantly increases at triglyceride levels above 500 mg/dl. Both primary causes, including genetic disorders such as familial chylomicronemia, and secondary disorders of lipid metabolism, including diabetes, hypothyroidism, and pregnancy, could cause HTGP. The toxic levels of triglycerides that break into free fatty acids by pancreatic lipases are critical in pancreatitis pathogenesis. The lipotoxicity, in turn, causes systemic inflammation with further complications related to it. The clinical features of HTGP are similar to other pancreatitis causes, including abdominal pain, nausea, and vomiting. Usually, patients with HTGP tend to have worse outcomes compared to other causes. Due to too high levels of triglycerides, the serum becomes milky and causes an alteration in serum electrolytes levels, including pseudo-hyponatremia. The recommended treatment for HTGP is plasma apheresis as well as IV insulin infusion, and heparin, specifically for less worrisome patients. IV insulin potentially avoids the interventional complexities of apheresis. The usual treatment goal is to reduce the triglycerides to a safe level, and then further management is tailored to lifestyle modification and oral lipid reducing agents. Our case report explains how well insulin works in stable patients with severe pancreatitis and thus prevents associated morbidity and mortality.

## Introduction

The basic function of exocrine pancreas is to secrete a variety of digestive enzymes that usually gets activated in the duodenum in order to participate in the digestive function. Acute pancreatitis (AP) is one of the many complications of hypertriglyceridemia. In this condition the proteolytic enzymes released from the pancreas get activated spontaneously and quickly leading to auto digestive injury of the gland. Usually once the triglycerides (TG) get a level greater than 500 mg/dl the risk of pancreatitis is significantly increased [1,2]. Due to significant inflammation, a number of inflammatory cytokines are released, causing further oxidative stress, thus leading to intra-arterial thrombosis and hemorrhage eventually causing pancreatic necrosis.

## Case presentation

We present a case of a morbidly obese 17-year-old Hispanic man who arrived at the emergency department with a three-day of worsening epigastric and right upper quadrant abdominal pain associated with emesis. On arrival, he had a temperature of 99.8 °F, blood pressure of 140/57 mmHg, heart rate of 121 beats per minute, respiratory rate of 20, oxygen saturation of 94% at room air, and body mass index (BMI) of 40.37 kg/m^2^. He was obese on physical examination, with hyperpigmentation, velvety skin, and multiple skin tags around the neck area, significant central adiposity, and mild epigastric tenderness. Labs were notable for lipase of 458 mg/dl and blood glucose (BG) of 350 mg/dl. He had a milky blood sample prompting triglyceride (TG) level check, which was greater than 4000 mg/dl (Table 1).

**Table 1:** Levels of triglycerides from admission to discharge

Time (hours)	Triglycerides (mg/dl)
0	4000
12	3457
24	1482
36	1056
48	755
60	669
72	548

On further investigation it was found that he had a hemoglobin A1C of 10.3%. His urinalysis showed proteinuria and ketonuria, his computed tomography (CT) abdomen revealed acute pancreatitis and diffuse hepatic steatosis. Serum alcohol level was normal and CT abdomen did not show any gallbladder pathology, also he has denied any trauma to the abdomen. He was diagnosed with hypertriglyceridemia-induced pancreatitis and new- onset diabetes. Due to hemodynamic stability, insulin drip with a goal of TG less than 500 mg/dl and BG of 250 mg/dl was recommended instead of immediate plasmapheresis by nephrology services. While on insulin drip blood glucose was closely monitored to prevent hypoglycemia (Table 2).

**Table 2:** Blood glucose levels while on insulin infusion

Time (hours)	Blood Glucose (mg/dl)
9 pm	350
1 am	265
3 am	260
5 am	261
7 am	286
8 am	299
11 am	290
2 pm	427
3 pm	224
6 pm	227
9 pm	195
11 pm	148

Later, he got transferred to the intensive care unit (ICU) and evaluated by an endocrinology service for subcutaneous insulin transition. TG levels significantly improved within 12 hours (4000→1,482) as shown in the table above and transitioned to subcutaneous insulin at a 548 mg/dl TG level.

After three days, he was successfully discharged home from ICU with subcutaneous insulin and fenofibrate prescriptions. He was extensively counseled regarding life style modifications, together with weight reduction, healthy dietary habits and medication compliance. 

## Discussion

Acute pancreatitis is one of the many complications of hypertriglyceridemia. Hypertriglyceridemia-induced pancreatitis (HTGP) is relatively uncommon with an estimated incidence of up to 10% of all the cases [3]. The high TG level, in turn, causes damage to vascular endothelium and capillary plugging with activation of trypsinogen, the proteolytic enzymes from the pancreas leading to the gland’s auto digestive injury [4]. Usually, once the TGs are at a level greater than 500 mg/dl, the pancreatitis risk is significantly increased [5]. Patients with acute pancreatitis (AP) attributed to hypertriglyceridemia are at a greater risk for complications, and diabetics tend to worsen than the general population [5,6]. The very high levels of free fatty acids and TGs in the circulatory system set up a pro-inflammatory cascade. Uncontrolled diabetes causes lipid peroxidation, a critical event in acute pancreatitis’ pathogenesis related to hypertriglyceridemia. As per one of the studies comparing high versus low level of TG, there were 66 patients in the low-TG group and 78 patients in the high-TG group. There was no significant difference in the age, sex ratio, BMI and comorbidities between the two groups. The high-TG group had significantly higher levels of glucose than the low-TG group [7]. The incidences of local complications and mortality were significantly higher in the high-TG group than in the low-TG group [6,7]. Also, comparing alcoholic versus HTGP studies have shown a statistically significant increase in the number of patients receiving intensive care admission and receiving surgical interventions related to HTGP [8].

## Conclusions

This case highlights that early use of insulin therapy can potentially mitigate the need for plasmapheresis. Myriad complications have been associated with plasmapheresis when employed in patients with HTGP. Insulin therapy can also prevent AP complications, including acute respiratory distress syndrome and renal failure. Often the insulin therapy gets overlooked in the context of potential ICU admission for close monitoring.

Given the safe, efficacious, and cost-effective way to rapidly reduce TG, we recommend prompt TG levels be drawn in patients presenting without gallbladder or alcohol-related pancreatitis and early initiation of insulin drip in those patients with TG above 1000 mg/dl. The risk of complications reduces once TG levels fall below 500 mg/dl, and further management is dependent on lifestyle modifications, including dietary habits, oral lipid-lowering agents, and optimal control of hyperglycemia in diabetic patients.
